# Non-Transgenic CRISPR-Mediated Knockout of Entire Ergot Alkaloid Gene Clusters in Slow-Growing Asexual Polyploid Fungi

**DOI:** 10.3390/toxins13020153

**Published:** 2021-02-16

**Authors:** Simona Florea, Jolanta Jaromczyk, Christopher L. Schardl

**Affiliations:** 1 Department of Plant Pathology, University of Kentucky, Lexington, KY 40546, USA; sflor2@uky.edu; 2 Computer Science Department, University of Kentucky, Lexington, KY 40546, USA; jolan-ta.jaromczyk@uky.edu

**Keywords:** CRISPR/Cas9, non-transgenic engineered fungi, genome editing, genome sequencing, MinION, nanopore, secondary metabolites

## Abstract

The *Epichloë* species of fungi include seed-borne symbionts (endophytes) of cool-season grasses that enhance plant fitness, although some also produce alkaloids that are toxic to livestock. Selected or mutated toxin-free endophytes can be introduced into forage cultivars for improved livestock performance. Long-read genome sequencing revealed clusters of ergot alkaloid biosynthesis (*EAS*) genes in *Epichloë coenophiala* strain e19 from tall fescue (*Lolium arundinaceum*) and *Epichloë hybrida* Lp1 from perennial ryegrass (*Lolium perenne*). The two homeologous clusters in *E. coenophiala*—a triploid hybrid species—were 196 kb (*EAS*1) and 75 kb (*EAS*2), and the *E. hybrida EAS* cluster was 83 kb. As a CRISPR-based approach to target these clusters, the fungi were transformed with ribonucleoprotein (RNP) complexes of modified Cas9 nuclease (Cas9-2NLS) and pairs of single guide RNAs (sgRNAs), plus a transiently selected plasmid. In *E. coenophiala*, the procedure generated deletions of *EAS*1 and *EAS*2 separately, as well as both clusters simultaneously. The technique also gave deletions of the *EAS* cluster in *E. hybrida* and of individual alkaloid biosynthesis genes (*dmaW* and *lolC*) that had previously proved difficult to delete in *E. coenophiala*. Thus, this facile CRISPR RNP approach readily generates non-transgenic endophytes without toxin genes for use in research and forage cultivar improvement.

## 1. Introduction

A few species of filamentous fungi have been genetic models of choice since the 1950s due to their haploid growth stage, facile sexual cycles, abundant sporulation, rapid growth and, with time, large repertoires of mutants and molecular transformation systems. However, given the importance of fungi in medicine, agriculture, and ecosystems, considerable efforts have been invested over several decades to establish molecular transformation and targeted mutation systems for a much broader range of species. These include the *Epichloë* species (family Clavicipitaceae, order Hypocreales), which are systemic, constitutive, and often seed-transmitted symbionts (endophytes) of cool-season grasses (Poaceae, subfam. Poöideae), and which are capable of producing a panoply of bioprotective alkaloids [1,2,3]. However, features of important *Epichloë* species that present special difficulties for genetic experimentation include growth rates far slower than model fungi, sparse sporulation, limited availability of selectable markers, and for many, diploid or triploid genomes and the lack of a sexual cycle [2,4]. 

The development of CRISPR technologies has opened the door to facile gene inactivation, removal, or even replacement for a wide range of organisms, including filamentous fungi [5,6]. Initially, the application of CRISPR in eukaryotes involved transformation or transfection with a gene construct expressing the Cas9 double-strand DNase and another construct to transcribe guide and tracrRNAs to direct the activity of Cas9 to the target sites. In *Aspergillus* species, Nødvig et al. [7] developed a system based on a single plasmid harboring a chimeric RNA guide and the *cas*9 gene under fungal promoters, along with a marker gene required for fungal selection. More recently, Cas9-sgRNA (single guide and tracrRNA) ribonucleoprotein complexes (RNPs) have been employed in a wide range of fungi [5,6]. 

In an example of the RNP approach, targeted mutations have been introduced into the genome of the fungus *Pyricularia oryzae* [8], which is among the most important fungal pathogens globally impacting cereal grain production. The fungus was transformed simultaneously with the RNPs to mutate the target gene and others to generate mutant genes that are positively selectable. This strategy is based on the presumption that those nuclei in which the genes are converted to their selectable forms are also those most likely to have taken up the RNP and consequently mutate the target gene as well. 

We use *Epichloë* spp. as exemplars of particularly difficult but important fungi for targeted genetic modification. *Epichloë coenophiala* is widespread as a seed-borne endophyte of the highly popular pasture, forage, and turf grass, *Lolium arundinaceum* (= *Schedonorus arundinaceus = Festuca arundinacea*; tall fescue), although its existence was unsuspected in the first decades of widespread propagation of the grass during the mid-20th century. The fungus provides important benefits that translate to enhanced stand longevity and productivity and improved tolerance of drought and other stresses [9,10], and it is capable of producing up to four different classes of alkaloids that protect the grass hosts against invertebrates [2,11]. Unfortunately, the strains of *E. coenophiala* that have been unwittingly co-propagated with tall fescue, and which remain dominant in much of the cool-season pasturelands, produce ergovaline, which is an ergot alkaloid of the highly toxic ergopeptine type [12,13,14]. Levels of ergovaline tend to be very low, but they are often sufficient to at least cause reproductive problems and reduce livestock health and productivity. For the same reason, cultivars of *Lolium perenne* (perennial ryegrass) with *Epichloë hybrida* [15] strain Lp1 were pulled from the market in 1992 after it was determined that they had toxic levels of ergovaline [16,17]. A study including deletion of the dimethylallyltryptophan synthase gene (*dmaW*) from *E. hybrida* Lp1 and its subsequent complementation with the ortholog from *Claviceps fusiformis* [18] has demonstrated that *dmaW* is essential for ergot alkaloid biosynthesis [19]. There is a potential to develop and deploy such genetically altered strains of *Epichloë* species in forage cultivars because the fungus can be cultured, manipulated, and reintroduced to produce new, stable symbioses with forage and pasture cultivars of tall fescue. However, it is desirable and perhaps essential that such modifications should not involve integration of any foreign gene in the genome, which is a requirement that makes the CRISPR RNP approach especially attractive.

The endophyte *E. coenophiala* is a particularly difficult system for genetic inquiry because of its slow growth [20] and the fact that it is a triploid interspecific hybrid [21]. Two of its three ancestors were ergovaline producers, so *E. coenophiala* has two homeologous copies of the ergot alkaloid biosynthesis (*EAS*) gene clusters [2]. An effort to delete the key gene *dmaW*2 by marker exchange mutagenesis with a hygromycin B-resistance gene (loxP-flanked *hph*) was a particular *tour de force* in which over 1500 transformants were screened and the frequency of homologous gene replacement was 0.2% [22]. Once a ∆*dmaW*2 mutant was obtained and reintroduced into host plants, there was essentially no effect on ergovaline production because of the presence of its homeologue *dmaW*1. Furthermore, although the selectable marker was readily removed from the ∆*dmaW*2 mutant by transformation with a plasmid for the transient expression of Cre recombinase [22], the resulting marker-free mutants consistently had lost the ability to establish stable symbiosis with tall fescue (unpublished results).

Natural mutants of *Epichloë* species with deleted or inactivated alkaloid biosynthesis genes consistently have inactivated the entire set of genes for downstream steps [23,24,25]. Whether or not this relates to the host incompatibility of the aforementioned marker-free ∆*dmaW*2 mutants, the nature of these natural variants suggests that there is selection against expression of enzymes that, due to loss of upstream genes, no longer have access to their normal substrates. Therefore, we consider the most prudent approach to generating ergot alkaloid-negative mutants to be the deletion of both *EAS* clusters in their entirety. 

After genome sequencing revealed the subterminal location of the *EAS*1 cluster in *E. coenophiala* isolate e19, we devised a new approach to replace that cluster with a telomere-repeat array [24]. Since the homeologous cluster, *EAS*2, has an inactivating mutation in a late-pathway gene, *lpsB*2, the resulting “*EAS*1-knockoff” mutant produced only two early products of the ergot alkaloid pathway, chanoclavine and ergotryptamine. The technique employed transient expression of antibiotic resistance conferred by an *hph* gene positioned in the vector to be lost subsequently by breakage of the integrated DNA at the introduced telomere repeat array. The success of this approach suggested that transient antibiotic selection could be used in other approaches for mutation. In this study, this strategy is applied to CRISPR-based deletion of both *EAS* clusters as well as individual genes.

Both for research and for the practical aim of completely eliminating production of all ergot alkaloids from an agriculturally important grass symbiont, we have chosen to adapt a Cas9-sgRNA RNP approach to entirely eliminate both the 196-kb *EAS*1 cluster and the 75-kb *EAS*2 cluster. Here, we describe the success of that effort and follow-up experiments to demonstrate the facile nature of our approach and its broader applicability, opening the door to a wide range of non-transgenic manipulations of even slow-growing, asexual, polyploid fungi.

## 2. Results

### 2.1. Assembly of E. coenophiala and E. hybrida Genome Sequences Including Nanopore Data

The genome of *E*. *coenophiala* e19 wild-type strain was previously sequenced by a combination of pyrosequencing (Roche) and Sanger sequencing of fosmid-cloned ends [24]. Due to its triploid hybrid nature, the genome is complex and has been difficult to assemble, especially across repetitive regions. To assess the potential of Oxford Nanopore technology to improve the genome data and assembly, the *E*. *coenophiala* e19 genome was sequenced using the portable DNA sequencer MinION. The MaSuRCA v. 3.4.1 de novo assembly was manually curated to give 216 scaffolds with the contig sizes varying from 1915 to 12,291,650 bp with an N50 of 1,403,312 bp (Supplementary Table S1; GenBank accession number JAFEMN000000000). The estimated genome size was 104.2 Mb. The 196.2 kb complete sequence of the *EAS*1 cluster was identified on a 676 kb scaffold that ended with a telomere repeat array, and the 75.2 kb *EAS*2 cluster was identified on a 2.6 Mb scaffold sequence where it was flanked by housekeeping genes. Both clusters had the 11 known ergot alkaloid biosynthesis genes similarly arranged and oriented, and the difference in their sizes was due to lengths of AT-rich noncoding regions consisting mainly of repeats.

The *E. hybrida* Lp1 genome was sequenced using several sequencing platforms (Supplementary Figure S1) and the assembled data generated 158 scaffolds with the contig size varying from 9259 to 8,313,425 bp, with an N50 of 2,103,505 bp, and estimated total genome size of 79.9 Mb sequence (GenBank accession number JAFEKR000000000). The 83.2-kb *EAS* cluster was located on a 6.7 Mb scaffold.

### 2.2. Deletion of Ergot Alkaloid Biosynthesis Gene Clusters from the E. coenophiala Genome

**Figure 1:**
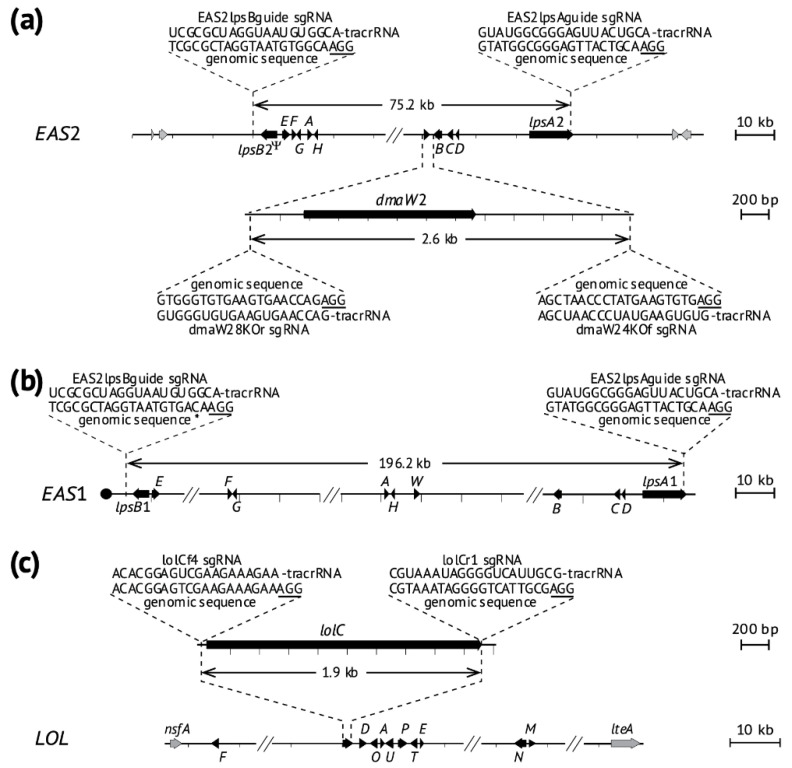
Maps of genes and gene clusters in *E. coenophiala* e19 indicating target locations and sequences of the single guide RNAs (sgRNAs) that direct cleavage by modified Cas9 nuclease. On each map, the AGG (underlined) protospacer adjacent motifs (PAM) required for Cas9 nuclease to generate double-strand break at the target site is 3′-adjacent to the 20-nucleotide target DNA sequence, and the trans-activating crispr RNA (tracrRNA) segment is a 67 nt sequence that interacts with Cas9. Black box-arrows indicate *EAS* or *LOL* genes, which are labeled with the full name or an abbreviation with the last letter of the gene name or with *B* for *cloA*. Hash marks on the *EAS* maps indicate long stretches of noncoding sequences. (**a**) Ergot alkaloid biosynthesis gene cluster *EAS*2 with the *dmaW*2 region magnified. The sgRNA sequences for *EAS*2 deletion match genomic sequences outside but near the 3′ end of the *lpsB*2 pseudogene and within but near the 3′ end of *lpsA*2. The sgRNA sequences for *dmaW*2 deletion match flanking intergenic regions. (**b**) Ergot alkaloid biosynthesis gene cluster *EAS*1. The sgRNA guides are the same as those for *EAS*2, and the sequence flanking *lpsB*1 has a single mismatch to the sgRNA (asterisk). (**c**) Loline alkaloid biosynthesis gene cluster *LOL* with *lolC* magnified.

**Figure 2:**
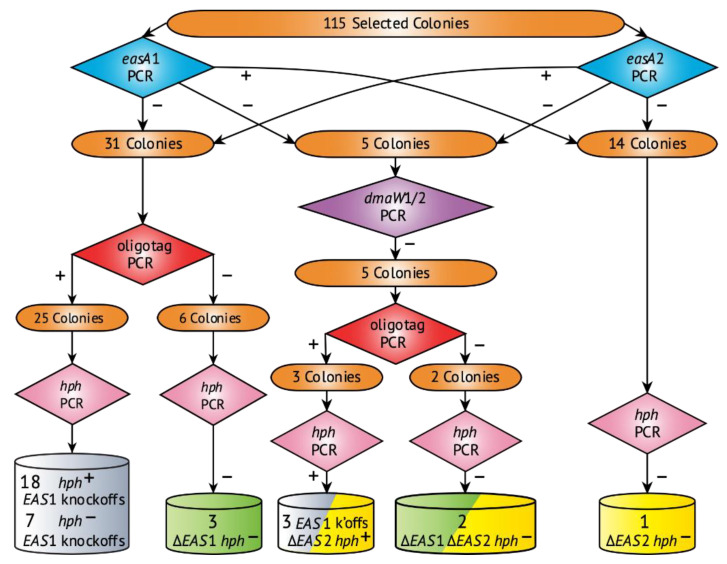
Graphic summary of the screening strategy and results for ergot alkaloid biosynthesis (*EAS*)*-*cluster deletions in *E. coenophiala* e19. Cylinders represent mutants identified as *EAS*1 knockoffs by chromosome-end deletions [24] (gray), Cas9-mediated *EAS*1 deletions (green; ∆*EAS*1), and Cas9-mediated *EAS*2 deletions (yellow; ∆*EAS*2). Cylinders with two colors represent losses of both *EAS*1 and *EAS*2 gene clusters in the same mutants.

After *E. coenophiala* e19 protoplasts were treated simultaneously with the *EAS*2-directed RNPs with specific sgRNAs (Figure 1 and Table 1) and the plasmid pKAES329 with a fungal-active *hph* gene, 115 hygromycin B-resistant colonies were recovered and subsequently single-spore isolated on plates without selection. DNA was extracted and subjected to a series of PCR screens (Figure 2 and Supplementary Figure S1) with primers designed for identification of the expected deletion mutants (Table 2). The first screen with primers specific for *easA*1 and *easA*2 indicated 31 colonies lacking only *easA*1, 14 lacking only *easA*2, and five lacking both *easA*1 and *easA*2. As a further check if both *EAS*1 and *EAS*2 clusters were absent in the last five transformants, they were subjected to a second PCR screen with primers targeting a common region in the two *dmaW* homeologs, and all five tested negative for both (Figure 2 and Supplementary Figure S1). 

Since the pKAES329 plasmid used to transiently select transformants carried a truncated fragment of *lpsA*1, and there was a single-base mismatch of the EAS2lpsBguide to the *EAS*1 locus target site (Figure 1), we expected that the loss of the *EAS*1 cluster in some mutants might be due to a homologous recombination of the *lpsA*1 sequence followed by chromosome-end knockoff, as was previously accomplished using the same plasmid [24], rather than by Cas9-mediated deletion. To check this possibility, 31 colonies that were negative for *easA*1 and another five that were negative for both *easA*1 and *easA*2 were tested by PCR with primers targeting a 223 bp fragment spanning from the oligotag into the truncated *lpsA*1 gene on pKAES329, which was a sequence that was expected to be present in such knockoff mutants [24] but to be absent from mutants induced only by CRISPR. The result was negative for six of the mutants that lacked only *easA*1 and two that lacked both *easA* genes, suggesting that the loss of *EAS*1 in those mutants was due to Cas9-catalyzed cleavage. The other mutants, which were positive for the oligotag, were not investigated further. The CRISPR-mediated cluster deletions were designated ∆*EAS*1, ∆*EAS*2 and ∆*EAS*1 ∆*EAS*2 (Figure 2).

In addition to generating strains completely lacking ergot alkaloid genes, the goal was to avoid integration of any foreign gene in the genome of the modified strains. A PCR test for *hph* identified two ∆*EAS*1 ∆*EAS*2 mutants (designated e7801 and e7802) and one ∆*EAS*2 mutant (designated e7803) that were marker-free (Figure 2 and Supplementary Figure S1).

### 2.3. Deletion of dmaW2

*Epichloë coenophiala* e19 is a triploid interspecific hybrid with orthologous *EAS*1 and *EAS*2 gene clusters that can largely complement each other’s ergot alkaloid-biosynthesis gene mutants [22,24]. We previously eliminated the telomere-linked *EAS*1 cluster to generate strain e7480 [24]. This “knockoff” mutant retained the *EAS*2 cluster with most functional ergot alkaloid biosynthesis genes including *dmaW*2, which encodes the enzyme for the first determinant step in the pathway [19], and which has proven exceptionally difficult to eliminate by marker-exchange homologous recombination [22]. Therefore, *dmaW*2 in e7480 was targeted to test if the use of CRISPR RNP technology might provide more efficient editing of the locus. Protoplasts of e7480 were simultaneously treated with the RNP mixture and pKAES329; then, they were regenerated on medium with hygromycin B to obtain a total of 318 selected colonies. Then, these were propagated without selection, and their DNA was screened by PCR for *dmaW*2 to identify 54 putative ∆*dmaW*2 mutants (Supplementary Figure S1). Screening those for *hph* indicated 50 out of the 54 putative mutants that also lacked the selection marker, which could have occurred either because the marker was expressed without plasmid integration or because of recapitulation of the chromosome-end knockoff [24] whereby the plasmid integrated at the *lpsA*1 site and was subsequently lost by breakage within the introduced telomere-repeat array. Positive controls were PCR screens with primers specific for *easA*2 and the oligotag and, as expected, all 50 putative marker-free mutants tested positive for both. Moreover, since the *EAS*1 cluster is missing in the e7480 genome, the PCR test for *easA*1 was negative for all the samples. Two of the ∆*dmaW*2 mutants were designated e7799 and e7800 (Table 3).

### 2.4. Deletion of the EAS Cluster in E. hybrida Lp1

*Epichloë hybrida* is a diploid interspecific hybrid that inherited, from its *Epichloë festucae* ancestor, an *EAS* cluster [15] similar to *EAS2* of *E. coenophiala*. The sgRNAs designed for deletion of the *EAS* clusters in e19 were used in an attempt to delete the *EAS* cluster in *E. hybrida* Lp1. The transformation plasmid providing transient hygromycin B resistance was pKAES328, which has the fungal-active gene *hph* but differs from pKAES329 in lacking an *lpsA*1 fragment [24]. Following the same PCR procedures as mentioned above for *E. coenophiala* (Supplementary Figure S1), 16 *E. hybrida* colonies were screened, out of which one (designated e7806) was a putative marker-free ∆*EAS* mutant (Table 3).

### 2.5. Deletion of lolC in E. coenophiala e19

Genomic analysis of *E. coenophiala* e19 indicated a single cluster, designated *LOL*, with the loline alkaloid biosynthesis genes. The *lolC* gene has been suggested to encode the enzyme for the first step in the loline alkaloid pathway and its role has been tested previously by RNA interference (RNAi) in *Epichloë uncinata,* resulting in significantly reduced loline-alkaloid production [26]. To further evaluate the general utility of the CRISPR RNP technology in *E. coenophiala,* sgRNAs were designed and used for the deletion of *lolC*. Following protoplast treatment with the RNP mixture followed by initial selection with hygromycin B, a total of 185 colonies were screened with *lolC*-specific primers to identify 11 that tested negative for the gene (Supplementary Figure S1). These putative ∆*lolC* mutants were further screened with primers for *hph,* identifying two marker-free mutants designated e7804 and e7805 (Table 3).

### 2.6. Genome Analysis of the CRISPR-Derived Mutants 

A de novo genome sequence assembly was performed for each deletion mutant in Table 3. The dataset size (in bases), number of reads (average length 130 bp), genome coverage, and assembly quality metrics for each are presented in Supplementary Table S2. In every case, the sequences were consistent with the PCR results regarding the gene losses due to Cas9 nuclease, absence of the marker gene, and absence of the oligotag sequence. Moreover, to check if any plasmid sequences were present in the deletion mutants, the reads were mapped against the pKAES328 and pKAES329. None of the mutants had sequences from those plasmids. 

**Figure 3:**
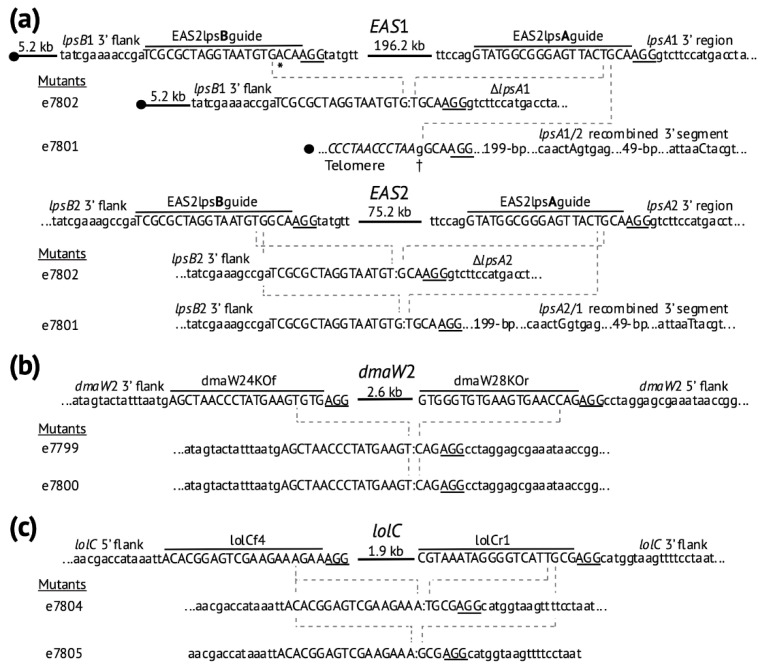
CRISPR-induced deletions in *E. coenophiala* e19 as deduced from genome sequencing. Targets of the sgRNAs (Table 1) are indicated, adjacent PAM sites (AGG in all cases) are underlined, and cleavage sites are assumed to have been 3 bp or 4 bp 5′ of the PAM sites. Colons (:) indicate joined ends. (**a**) Deletions and recombination at *EAS* gene loci. Filled circles indicate telomere repeat arrays of (CCCTAA)_n_ near *EAS*1, and an asterisk (*) indicates a mismatch between EAS2lpsBguide sgRNA and its target near the *EAS*1 gene cluster. In the ∆*EAS*1 locus of e7801 a single base-pair addition is indicated (†) just inside the new telomere (italic text and filled circle). In e7801, single-nucleotide polymorphisms within the recombined segments labeled *lpsA*1/2 and *lpsA*2/1 are indicated as capital letters. (**b**) CRISPR-mediated deletion of the *dmaW*2 gene for the first step in ergot alkaloid biosynthesis. (**c**) CRISPR-mediated deletion of the *lolC* gene for the presumed first step in loline alkaloid biosynthesis.

Interestingly, almost all of the gene deletions resulted from the cleavage and flawless rejoining of the two flanking ends with no other sequence changes (Figure 3). Positions of the cleavage by Cas9 in the e7801 and e7802 ∆*EAS*1 ∆*EAS*2 mutants varied between the strains but also between the two clusters in the same strain. For instance, in e7802, the cleavage at the *EAS*1 cluster near the *lpsB*1 locus was inferred to be 3-bp upstream of the PAM site, even though the position had a mismatch between the sgRNA and *EAS*1 target site, and 4-bp upstream of the PAM site in *EAS*2 where the sgRNA was an exact match to the target sequence. Furthermore, the cleavage near *lpsA*1 was 4-bp upstream of the PAM site but 3-bp upstream of the PAM site near *lpsA*2. Among the six sequenced mutants with 15 Cas9-mediated cleavage sites in all, 11 cleavages were 3-bp from the PAM site and four (27%) were 4-bp from the PAM site.

The changes at both *EAS* clusters in e7801 differed from those in e7802 in several interesting ways. In addition to the different cleavage sites mentioned above, e7801 (but not e7802) had undergone a reciprocal recombination event between the two *EAS* clusters, which was likely triggered by the Cas9-induced double-strand breaks in the orthologous positions of the two *lpsA* genes. Furthermore, in e7801, the cleaved end of the *EAS*1 cluster was linked to a new telomere repeat array, from which it was separated by only a single base pair. In contrast, both the *EAS*1 and the *EAS*2 clusters of e7802 were deleted with the flanking sequences joined.

A BLAST (basic local alignment search tool) search against the *E. hybrida* e7806 mutant genome with sequences for the *EAS* cluster and *hph* gene indicated their absence, which was consistent with the previous PCR results. The Cas9-mediated cleavage occurred 3 bp upstream from the PAM site near *lpsB* and 4 bp upstream from the PAM site in *lpsA*. The junction generated by joining the cleaved ends was identical to the junction in the deleted *EAS*2 locus of mutant e7801 shown in Figure 3. 

The *E. coenophiala* mutants e7999 and e7800 had lost *dmaW*2 as indicated by the PCR screen and validated by their sequenced genomes. Since the cleavages induced by Cas9 occurred 3 bp upstream of PAM sequence for the dmaW24KOf and dmaW28KOr sgRNA target sites, both ∆*dmaW*2 mutants had identical junctions with no indels (Figure 3).

The genome sequence of *E. coenophiala* ∆*lolC* mutants e7804 and e7805 also confirmed that no other foreign DNA was integrated into the genome. When comparing the *lolC* deletion site in the two mutants, it appeared that Cas9 had generated cleavages 3 bp upstream from PAM site at all sites except for the lolCr1 sgRNA target site of e7404, for which the cleavage occurred 4 bp upstream of the PAM site. The rejoining of the ends was perfect without introduction of indels or any sequence changes (Figure 3).

### 2.7. Inoculation Efficiency

Endophyte infection determined by tissue-print immunoblot indicated that all CRISPR-mediated deletion mutants established symbiotic relationships with the plant at infection rates similar or higher than those typically seen for wild-type strain inoculations (Table 4).

## 3. Discussion

We have demonstrated that a CRISPR/Cas9 technology can be applied to polyploid *Epichloë* species for the precise removal of individual genes or gene clusters, and even the simultaneous removal of a pair of large clusters (196 kb and 75 kb). Specifically, the method utilized RNPs—consisting of sgRNAs and Cas9 protein that was translationally fused with two NLRs—which were introduced into the fungus by cotransformation with a transiently selected antibiotic resistance gene. There are two obvious advantages to this approach. One is that more than one gene or genome region can be deleted in a single procedure, and the other is that the procedure leaves no selectable marker or transgenes in the genome. The absence of the selectable marker in the final product allows for its reuse to eliminate additional genes or to reintroduce genes for complementation analysis, and it also addresses regulatory and public concerns about the use of transgenic organisms in applied research and agriculture. 

We previously constructed a plasmid (pKAES329) to transiently integrate a selectable antibiotic-resistance gene (*hph*) at a chromosome end [24], and here, we have combined that approach with the RNP approach and found it to be successful and facile. However, we also questioned whether a transient integration of the marker was even necessary. We show here that marker integration is unnecessary by demonstrating the simultaneous elimination of two long gene clusters (*EAS*1 and *EAS*2) by treatment with the appropriate RNP mixtures together with a plasmid that, without integrating into the genome, provided expression of the selectable marker. 

In this study, we did not test whether even transient marker selection was needed. However, we previously used the transformation of *Epichloë* species without selection to delete a loxP-flanked *hph* gene by transient expression of the Cre-recombinase gene [22]. Although successful, the screen was tedious and time consuming, resulting in a 0.5–2.1% frequency of Cre-mediated deletions among the unselected colonies. In contrast, here, we report that plasmids mixed with the RNPs provided for temporary selection of a limited number of initially antibiotic-resistant transformants. From those, mutants with the target deletions were readily identified by PCR screens and a high proportion were marker free (18–100% depending on the experiment). Therefore, although integration of the plasmid-borne selectable marker was not required, our results suggest that that its inclusion in the transformation mixture aided in selection of the RNP-transformed isolates, including those with the desired deletions. 

In targeting the *EAS*1 gene cluster, a mismatch 3 bp upstream of the PAM site between the EAS2lpsBguide and its target genomic sequence near *lpsB*1 did not prevent cleavage at this position. The mismatch at a single-nucleotide polymorphism between the sites near *EAS*1 and *EAS*2 (the sgRNA sequence was based on the latter) was in the “seed” region but not part of the “core” region for sgRNA-directed Cas9 cleavage [27]. Since deletion of the 196-kb *EAS*1 region was achieved with cleavage at the mismatch position, a precise match to the target was evidently not required.

Two additional genome alterations were observed in one of the ∆*EAS*1 ∆*EAS*2 mutants. One was the introduction of a telomere at the cut site near *EAS*1 in one of the mutants. Interestingly, that cut was repaired very simply with the addition of a single base pair followed by a telomere array. The other alteration in the same mutant was a recombination between the remnants of *lpsA*1 and *lpsA*2, presumably moving the genes near *lpsA*2 from genomic locations far from a telomere to close proximity to the newly generated telomere. Whether that alteration affects the expression of those genes may be an interesting topic for future inquiry.

Our results demonstrate the high efficiency of the CRISPR/Cas9 technology while not indicating any size limit for genome segments that can be efficiently and precisely deleted. The frequency of *dmaW*2 deletion was 17%, compared to only 0.2% previously obtained for marker exchange mutagenesis of the same gene in the same *E. coenophiala* strain [22]. In addition, the frequency of the 75-kb *EAS*2 deletion (5.2%) was not dramatically less than that of the 2.6-kb *dmaW*2 deletion (17%) and was very close to that of the 1.9-kb *lolC* deletion (5.9%). Conceivably, size limits might be imposed by boundaries of chromatin states, so it is worth considering whether or not the clean deletions of large genome regions are more likely when those genes are part of a coordinately regulated cluster. 

Though often characterized as “error-prone”, non-homologous end joining (NHEJ) frequently occurs without error. A study in mouse cell lines reported 70% precise NHEJ events after Cas9-mediated cleavage [28]. Many target sites also exhibited approximately 10% 1-bp and 2-bp templated insertions. Insertions of 1 bp following Cas9 cleavage and NHEJ have also been described in *Saccharomyces cerevisiae*, with 74–100% apparently being templated, implying that in those cases, Cas9 cleavage left a staggered (5′-overhanging) end that was filled in by DNA polymerase 4 [29]. In our study, all of the eight sequenced junctions appeared to be precisely joined, but four of those had one or the other end cleaved 4 bp from the PAM site. In those four cases, the result was similar to the common 1-bp templated insertions observed by Lemos et al. [29], so it is reasonable to speculate that they also resulted from staggered cleavage and end-repair.

As commercial sources have recently made Cas9-NLS fusion proteins and synthetic sgRNAs available, a variety of approaches that include Cas9-sgRNA RNPs have been employed in fungi. Most involve the integration of a stable selectable marker [30] or simultaneous generation of a selectable mutation [8]. Khan et al. [31] report using the Cas9-sgRNA RNP system without selection to target the *TOX3* effector gene in *Parastagonospora nodorum*, with all of the six analyzed “transformants” exhibiting mutations at the repair site. This result contrasts with ours in that we found no mutations other than the deletions resulting from rejoining of the cleaved ends and, in approximately half of the junctions, an apparent 1-bp templated insertion. Thus, applications of this technology in different fungal systems may give substantially different outcomes.

## 4. Conclusions

We report five findings with regard to the application of Cas9-sgRNA RNP technology for deleting DNA segments in *Epichloë* species. First, transient selection for an antibiotic resistance gene included in the transformation mix allowed for the efficient identification of deletion mutants. Second, the deletions were precisely repaired by NHEJ (sometimes with templated 1-bp insertions). Third, even large segments up to 196 kb in our tests were efficiently deleted. Fourth, simultaneous deletions of two large DNA segments with entire biosynthetic gene clusters were obtained. Fifth, the deletion mutants retained their compatibility as grass symbionts. These results highlight the range of non-transgenic manipulations of even slow-growing, asexual, polyploid fungi that can be undertaken with this CRISPR RNP approach. The application to *Epichloë* species provides for tailored genotypes to use in turf and forage, for example to generate elite lines of livestock-friendly cultivars.

## 5. Materials and Methods 

### 5.1. Biological Materials

The wild-type *Epichloë coenophiala* strain e19 [= American Type Culture Collection (ATCC) 90664] from tall fescue (*Lolium arundinaceum*) cv. Kentucky 31 [32], and the *E. coenophiala EAS*1-knockoff strain e7480 (=ATCC PTA-126679) [24] were maintained in tall fescue elite breeding line KYFA0601 [24]. The wild-type *Epichloë hybrida* Lp1 (=ATCC TSD-66) strain from perennial ryegrass (*Lolium perenne*) [33] was maintained in perennial ryegrass cv. Rosalin [19]. The fungi were isolated from these symbiotic plants and grown as described in Florea et al. [34]. 

### 5.2. Miscellaneous Molecular Methods

Plasmid DNA was isolated from bacterial cultures by use of the ZR Plasmid Miniprep-Classic kit (Zymo Research, Irvine, CA, USA). Fungal DNA was isolated from fresh mycelium by use of the DNeasy 96 Plant Kit (Qiagen, Valencia, CA, USA) and a Geno/Grinder 2000 (SPEX CertiPrep, Metuchen, NJ, USA) or by use of the ZR Fungal/Bacterial DNA MiniPrep kit (Zymo Research). The DNA quality and concentrations were assessed by NanoDrop and Qubit (ThermoFisher Scientific, Waltham, MA, USA). Plasmids pKAES328 and pKAES329, with a fungal-active hygromycin B-resistance gene (*hph*), are described in Florea et al. [24].

### 5.3. Nanopore Single-Molecule Sequencing of Genomic DNA

Genomes of wild-type *E. coenophiala* e19 and *E. hybrida* Lp1 were sequenced by a hybrid approach including Illumina, pyrosequencing, and single-molecule sequencing. For Oxford Nanopore (Oxford, UK) single-molecule sequencing, the fungi were grown on potato dextrose agar (PDA) (BD, Franklin Lakes, NJ, USA) plates topped with cellophane for ease of removal. High molecular weight (> 40 kb) DNA was extracted from young mycelium as described by the method of Al-Samarrai and Schmid [35] and recovered by spooling on glass rods. The Ligation Sequencing Kit 1D (SQK-LSK108) was used for library preparation. For each library, the end-repair and dA-tailing step was performed with the NEBNext End repair/dA-tailing kit (New England Biolabs, Ipswich, MA, USA) by incubating 100 μL Ultra II End-prep reaction buffer containing 3 μg of high molecular weight DNA and 6 μL of Ultra II End-prep enzyme mix for 20 min at 20 °C, then for 20 min at 65 °C. Then, the end-repaired DNA was mixed with 120 μL AMPure XP beads (Beckman Coulter, Pasadena, CA, USA) and incubated 5 min at room temperature. The beads were pelleted on a magnetic rack and the supernatant was discarded; then, they were washed twice with 200 μL of 70% ethanol and allowed to dry. The beads were resuspended in 31 μL of nuclease-free water and incubated for 10 min at room temperature; then, they were pelleted on the magnetic rack until the solution became clear. Then, the DNA solution was transferred into a new 1.5 μL Eppendorf loBind DNA tube, and the DNA concentration was determined using the Qubit. The adapter ligation reaction was performed with 50 μL NEB Blunt/TA Ligase Master Mix and 20 μL adapter mix added to 30 μL containing 2 μg of the End-prep-treated DNA and incubated for 10 min at room temperature. Then, the adapter-ligated DNA was added to 60 μL AMPure XP beads and incubated for 5 min at room temperature; then, it was pelleted on a magnetic rack, and the supernatant was discarded. The beads were resuspended in 500 μL adapter bead binding buffer (ABB), pelleted again on the magnetic rack for the removal of the ABB buffer, allowed to dry, and then resuspended in 15 μL elution buffer. After 10 min incubation at room temperature, the beads were pelleted once more on the magnetic rack to recover the DNA library in the supernatant. 

Oxford Nanopore FLO-MIN106 flowcells were primed with a mix consisting of 480 μL RBF (running buffer with fuel mix) and 520 μL nuclease-free water by loading 800 μL of the priming mix into the priming port and incubating at room temperature for 5 min. The flowcell priming was completed by lifting the SpotOn port cover and then loading the remaining 200 μL of priming mix through the priming port. Then, the 75 μL DNA library loading mix consisting of 12 μL DNA library mixed with 35 μL of RBF, 2.5 μL nuclease-free water, and 25.5 μL LLB (library loading beads, EXP-LLB001) was loaded on the flowcell via the SpotON port. Sequence runs were implemented with MinKNOW software version 3.1.0.30 with the NC_48Hr_e19_exp_Run_FLO-MIN106_SQK-LSK108 and NC_48Hr_Lp1_exp_Run_FLO-MIN106_SQK-LSK108 sequencing scripts, and with the live base calling turned off. The MinION FAST5 output files were processed using Guppy basecalling software ver. 2.2.2 supporting GPU processing with NVIDIA-SMI driver version 418.67 and CUDA version 10.1. Guppy was run as a singularity container on the University of Kentucky HPC system. 

### 5.4. Genome Sequence Assembly

Oxford Nanopore and Illumina HiSeq data (described above) were combined with Roche 454 GS FLX+ (454 Life Sciences, Branford, CT, USA) and paired-end Illumina MiSeq data described previously [24] in genome assemblies using MaSuRCA ver. 3.4.1 [36], which is a de novo assembler that combines the benefits of deBruijn graph and overlap-layout-consensus assembly approaches [37]. For Oxford Nanopore data, only the longest reads providing 25-fold genome coverage were included. 

### 5.5. Illumina HiSeq Sequencing of Genomic DNA

DNA libraries were prepared by use of the Nextera DNA library prep and index kits per the manufacturer’s reference guide (Epicentre Biotechnologies, Madison, WI, USA). Sequencing was carried out by Novogene Corporation Inc. (Sacramento, CA, USA) on the HiSeq platform with 2 × 150-cycle paired-end reads with 11 barcoded samples multiplexed on one lane (Illumina, San Diego, CA, USA). The data were evaluated for quality using FastQC v. 0.11.9 (https://www.bioinformatics.babraham.ac.uk/projects/fastqc/ (accessed on 20 October 2020)). Based on the FastQC results, the reads were trimmed, and the remnants of the adapters were removed using Trimmomatic v. 0.39 [38]. Duplicate reads were removed using Prinseq-lite v. 0.20.4 [39]. Assembly was performed with MaSuRCA v. 3.4.1. 

### 5.6. Design of sgRNA Molecules and Assembly of RNP Complexes

The sgRNAs were designed using the Benchling platform (https://www.benchling.com/ (accessed on 20 October 2020)), having the genome of *Claviceps purpure*a 20.1 set as reference in the guide parameter design. All the sgRNAs used in this study were designed upstream of an AGG protospacer adjacent motif (PAM) recognition site (Table 1). Cas9 nuclease typically cleaves the DNA 3-bp upstream of the PAM site [40]. The sgRNAs chosen to delete *EAS* clusters were exact matches to target sequences near the ends of the *EAS*2 cluster. One (EAS2lpsBguide) was 163 bp downstream of *lpsB*2, and the other (EAS2lpsAguide) was 718 bp upstream of the stop codon of *lpsA*2. The guide sequence in EAS2lpsAguide was also an exact match to the homologous sequence in *lpsA*1, but EAS2lpsBguide had a single mismatch located 3 bp from the PAM site at the sequence near *lpsB*1 (Figure 1). 

The *dmaW*2 sgRNAs were designed to recognize target sequences 431 bp upstream (dmaW28KOr) and 1055 kb downstream (dmaW24KOf) of the *dmaW*2 coding region and were an exact match to the genomic sequence at the *dmaW*2 locus. The *lolC* sgRNAs were designed to target sequences 140 bp upstream (lolCf4) of the start codon and 15 bp downstream (lolCr1) of the stop codon, and they were an exact match to the genomic sequence near the *lolC* gene (Figure 1). The *Streptococcus pyogenes* Cas9 nuclease fused with two nuclear localization signal motifs (Cas9-2NLS)*,* and the sgRNAs were purchased from Synthego Corp. (Redwood City, CA, USA). To form RNP complexes, 180 pmol of each sgRNAs was incubated 10 min at room temperature with 20 pmol Cas9-2NLS nuclease and nuclease-free water in 15 μL volume. For transformation, the RNP complexes were paired and mixed with the plasmid DNA, as indicated in Table 1.

### 5.7. Fungal Transformation and Selection

*Epichloë coenophiala* e19, its derivative e7480 [24], and *E. hybrida* Lp1 were transformed with the plasmid–RNP mixtures by a modification of previously described methods [24,41] as follows: Fungus was grown in potato dextrose broth (PDB) (BD, Franklin Lakes, NJ, USA) in an incubator shaker for 5–10 days at 22 °C and 200 rpm. The culture was transferred into 50 mL conical tubes and harvested by centrifugation at 5525× *g* for 20 min at 4 °C. The mycelium was resuspended in 20 mL osmotic medium (1.2 M MgSO_4_, 10 mM NaHPO_4_) containing an enzyme mixture consisting of 5 mg/mL Vinoflow FCE (Novozymes, Franklington, NC, USA), 5 mg/mL lysing enzymes from *Trichoderma harzianum*, 5 mg/mL Driselase Basidiomycetes sp., and 3 mg/mL bovine serum albumin (all from Sigma-Aldrich, St. Louis, MO, USA); then, it was incubated for 3 h on a rocker shaker at 30 °C. The remaining mycelial mass was removed from the protoplast suspension by passage through autoclaved Miracloth; then, it was transferred into 30 mL Corex tubes at 10 mL of the suspension in each, which was gently overlayed with 10 mL of ST solution (0.6 M sorbitol, 0.1 M Tris-Cl pH 7.4). To isolate the protoplasts, the tubes were centrifuged at 3329× *g* for 20 min. The protoplasts were rescued from the interface of the two solutions with a pipette and transferred into a tube containing 5 mL of STC solution (1 M sorbitol, 50 mM Tris-Cl pH 7.4, 50 mM CaCl_2_). The protoplasts in STC were pelleted in a centrifuge at 3329× *g* for 10 min. The supernatant was discarded, and the protoplast pellet was gently resuspended in 5 mL STC and pelleted again, and the supernatant was discarded. Finally, the protoplasts were gently resuspended in a small volume of STC, counted by microscopy with a hemocytometer, and diluted in STC so that 100 μL solution would contain at least 5 × 10^6^ protoplasts. 

Protoplasts were transformed using a modification of the polyethyleneglycol (PEG) method as described by Panaccione et al. [41]. The transformation mix was prepared by combining 15 μL of each preassembled RNP complex with 7–10 μg of *Mlu*I-linearized plasmid DNA that was previously incubated 30 min at room temperature with 10 μg of Lipofectin Transfection Reagent (ThermoFisher Scientific, Waltham, MA, USA). The PEG-amendment solution was prepared by mixing two parts of 60% (*w*/*v*) PEG 3350 with one part amendments (1.8 M KCl, 150 mM CaCl_2_, 150 mM Tris-HCl pH 7.4). The transformation was done in a sterile borosilicate tube by gently mixing 100 μL of protoplast suspension with 50 μL PEG-amendment solution and the DNA mix, and it was incubated on ice for 30 min. Then, 1 mL of PEG-amendment solution was added to the content of the tube and mixed by flicking the tube several times; then, it was incubated for 20 min at room temperature.

The treated protoplasts were plated on complete regeneration medium (CRM) [41], which contained per liter 304 g of sucrose, 1 g of KH_2_PO_4_, 1 g of NaCl, 0.46 g of MgSO_4_ -7H_2_O, 0.13 g of CaCl_2_-2H_2_O, 1 g of NH_4_NO_3,_ 1 g of yeast extract, 12 g of PDB powder, 1 g of peptone, 1 g of casein (acid hydrolysate), and 7 g of agarose (Sigma-Aldrich). Plates of 20 mL CRM bottom-agarose contained hygromycin B at concentrations calculated to give a final 50 μg/mL for *E. coenophiala* or 200 μg/mL for *E. hybrida*. Each aliquot (250 µL) of treated protoplast suspension was added to 7 mL of CRM prepared with low melting Sea Plaque Agarose (Lonza, Mapleton, IL, USA) and kept molten at 50 °C; then, it was immediately poured and distributed onto the surface of a CRM plate. The plates were incubated at 21 °C for 3–4 weeks, and then, the fungal colonies were transferred to PDA without hygromycin B for sporulation and single-spore isolation. 

### 5.8. Screening and Analysis of Deletion Mutants 

Putative transformants were subjected to three rounds of single-conidiospore isolation on PDA without selection; then, they were grown and maintained on PDA plates. DNA was extracted by use of the DNeasy 96 Plant Kit (Qiagen, Valencia, CA, USA). PCR primers were purchased from Integrated DNA Technologies (Coralville, IA, USA) and are listed in Table 2. PCR was performed in 25 μL reaction mixtures with 5–10 ng DNA template, 200 μM each dNTP, 0.2 μM each primer, 2.5 units AmpliTaq Gold, and AmpliTaq Gold PCR buffer with MgCl_2_ at 1.5 mM final conc. (Applied Biosystems, Foster City, CA, USA). Thermocycler conditions were 9 min at 94 °C, 35 cycles of 94 °C for 30 s, annealing temperature 59 °C for 30 s, 72 °C for 1 min, and then a final 7-min incubation at 72 °C.

Selected isolates were subjected to Illumina HiSeq DNA sequencing (see above) to check for the results of deletions and nonhomologous end joining at the target sites. The reads were also screened for sequences from transformation plasmids pKAES328 and pKAES329 by using Deconseq ver. 0.4.3 [42].

### 5.9. Establishment of Symbiota

To establish symbiotic relationships with host plants, wild-type *E. coenophiala* and mutants were surgically introduced into seedlings of tall fescue elite breeding line KYFA0601, and *E. hybrida* mutants were similarly introduced into seedlings of the perennial ryegrass experimental line GA66 [43] by the procedure described by Latch and Christensen [44] and modified by Chung et al. [45]. After seedlings developed at least three tillers, one tiller each was sacrificed and analyzed for presence of the fungus by tissue-print immunoblot with antiserum raised against *E. coenophiala* protein [46].

## 6. Patents

Pending: Schardl, C.L.; Florea, S.; Farman, M.L. Fungal chromosome-end knockoff strategy. US 2017 /0349899 Al, 2017.

## Figures and Tables

**Table 1:** sgRNA guides.

Scheme 1.	Target Site	PAM	Modified sgRNA Sequence ^1^
dmaW24KOf	*dmaW*2 3′-flank	AGG	A*G*C*UAACCCUAUGAAGUGUG
dmaW28KOr	*dmaW*2 5′-flank	AGG	G*U*G*GGUGUGAAGUGAACCAG
EAS2lpsAguide	*lpsA* 3′-region	AGG	G*U*A*UGGCGGGAGUUACUGCA
EAS2lpsBguide	*lpsB* 3′-flank	AGG	U*C*G*CGCUAGGUAAUGUGGCA
lolCf4	*lolC* 5′-flank	AGG	A*C*A*CGGAGUCGAAGAAAGAA
lolCr1	*lolC* 3′-flank	AGG	C*G*U*AAAUAGGGGUCAUUGCG

^1^ Asterisks indicate 2′ O-methyl analogs with 3′ phosphorothioate linkages.

**Table 2:** Primers used for PCR tests.

Primer Name	Target Gene(s) or Sites	Sequence
dmaW1&2f	*dmaW*1, *dmaW*2	GCAAAGACACTCCACCAGGAAGTT
dmaW1&2r	*dmaW*1, *dmaW*2	AGTTGCGGCGTTAATAGGCTCGTA
hph.4d	*hph*	GACCTGATGCAGCTCTCGGA
hph.3u	*hph*	TCGGCGAGTACTTCTACACA
RTeasA1f	*easA*1	ACAACTTTGGGCGACTGGG
RteasA1r	*easA*1	CCGTTGGTTGCAAGAAGATTGA
RteasA2	*easA*2	GCAGCTTTGGGCGACTGGA
RteasA2r	*easA*2	ACGTTGGTTGCAAGGAGATTGG
lolC-3a	*lolC*	GGTCTAGTATTACGTTGCCAGGG
lolC-5b	*lolC*	TCTAAACTTGACGCAGTTCGGC
oligoscreen(f)	Oligotag	GATGGCCTTTAAAGTCTACGTACTC
lpsAoligoR	*lpsA*1, *lpsA*2	ATATCATGGCAACATTCAGCGCAC

**Table 3:** Mutants confirmed by genome sequencing.

Mutant	Parental Strain	Genotype
e7799	e7480	EAS1-knockoff ∆*dmaW*2
e7800	e7480	EAS1-knockoff ∆*dmaW*2
e7801	e19	∆*EAS*1 ∆*EAS*2
e7802	e19	EAS1-knockoff ∆*EAS*2
e7803	e19	∆*EAS*2
e7804	e19	∆*lolC*
e7805	e19	∆*lolC*
e7806	Lp1	∆*EAS*

**Table 4:** Establishment of symbiosis of mutant endophytes with host plants.

Genotype Inoculated	Parental Strain	Plant Species	Positive/Total	Infection Rate %
∆*EAS*1 ∆*EAS*2	*Epichloë coenophiala* e19	*Lolium arundinaceum*	65/143	45
∆*EAS*2	*E. coenophiala* e19	*L. arundinaceum*	22/48	46
∆*EAS*	*Epichloë hybrida* Lp1	*Lolium perenne*	32/46	70
∆*dmaW*2	*E. coenophiala* e7480	*L. arundinaceum*	125/251	50
∆*lolC*	*E. coenophiala* e19	*L. arundinaceum*	25/81	31

## Supplementary Materials

The following are available online at https://www.mdpi.com/2072-6651/13/2/153/s1, Figure S1: PCR tests of putative CRISPR/Cas9-mediated deletion mutants, Table S1: Genome assembly statistics for wild-type *Epichloë* species, Table S2: Genome assembly statistics for CRISP/Cas9-mediated mutants.
