# Supplementary material for: Non-Transgenic CRISPR-Mediated Knockout of Entire Ergot Alkaloid Gene Clusters in Slow-Growing Asexual Polyploid Fungi
Source: Toxins (Basel). 2021 Feb 16;13(2):153. doi: 10.3390/toxins13020153 (PMC7922272; doi:10.3390/toxins13020153)
Supplement: Supplementary file 1 [file toxins-13-00153-s001.pdf]

Supplementary Materials: Non-Transgenic CRISPR-Mediated Knockout of Entire Ergot Alkaloid Gene Clusters in Slow-Growing Asexual Polyploid Fungi

Simona Florea, Jolanta Jaromczyk and Christopher L. Schardl

Table S1. Genome assembly statistics for wild-type *Epichloë* species.

Metric	*Epichloë coenophiala* e19	*Epichloë hybrida* Lp1
Genome assembly size (bp)	104,225,103	79,884,907
Scaffolds (no.)	216	158
Max. scaffold size (bp)	12,291,650	8,313,425
Min. scaffold size (bp)	1915	9259
N50 (bp)	1,403,312	2,103,505
L50 (no. of scaffolds)	18	10
N75 (bp)	684,515	887,067
L75 (no. of scaffolds)	45	27
Illumina reads (no.)	16,788,872	71,267,884
Illumina base calls (no.)	4,448,992,415	9,127,788,485
Ave. Illumina read length	265	128
Illumina coverage (fold)	43	114
Pyrosequencing reads (no.)	5,776,008	1,124,539
Pyrosequencing base calls (no.)	2,161,940,715	1,076,298,682
Ave. pyrosequencing read length	374	957
Pyrosequencing coverage (fold)	21	13
Nanopore reads (no.)	361,005	878,554
Nanopore base calls (no.)	2,111,083,110	2,779,076,273
Ave. nanopore read length	5848	3163
Nanopore coverage (fold)[1]	20	35
Total fold coverage	84	128

[1] Only the largest nanopore reads totaling 25-fold coverage were used in the assembly.

Table S2. Genome assembly statistics for CRISPR/Cas9-mediated mutants.

Mutant	Genome Assembly Size (bp)	Scaffolds (no.)	Max. Scaffold Length (bp)	N50 (bp)	Reads (no.)	Base Calls (no.)	Coverage (fold)
e7799	90,327,603	29,499	103,486	5611	36,650,711	9,411,821,892	104
e7800	85,819,038	30,898	89,851	4904	30,654,940	7,896,903,623	92
e7801	86,426,610	6978	203,325	26,484	40,964,917	10,469,527,658	121
e7802	88,355,357	31,299	117,788	5000	29,022,201	7,397,281,238	84
e7804	86,152,688	31,471	83,061	4802	34,372,962	8,842,724,973	103
e7805	89,955,890	29,760	99,576	5454	36,448,796	9,381,695,565	104
e7806	71,447,975	15,937	97,404	10,468	34,419,574	8,841,841,659	124

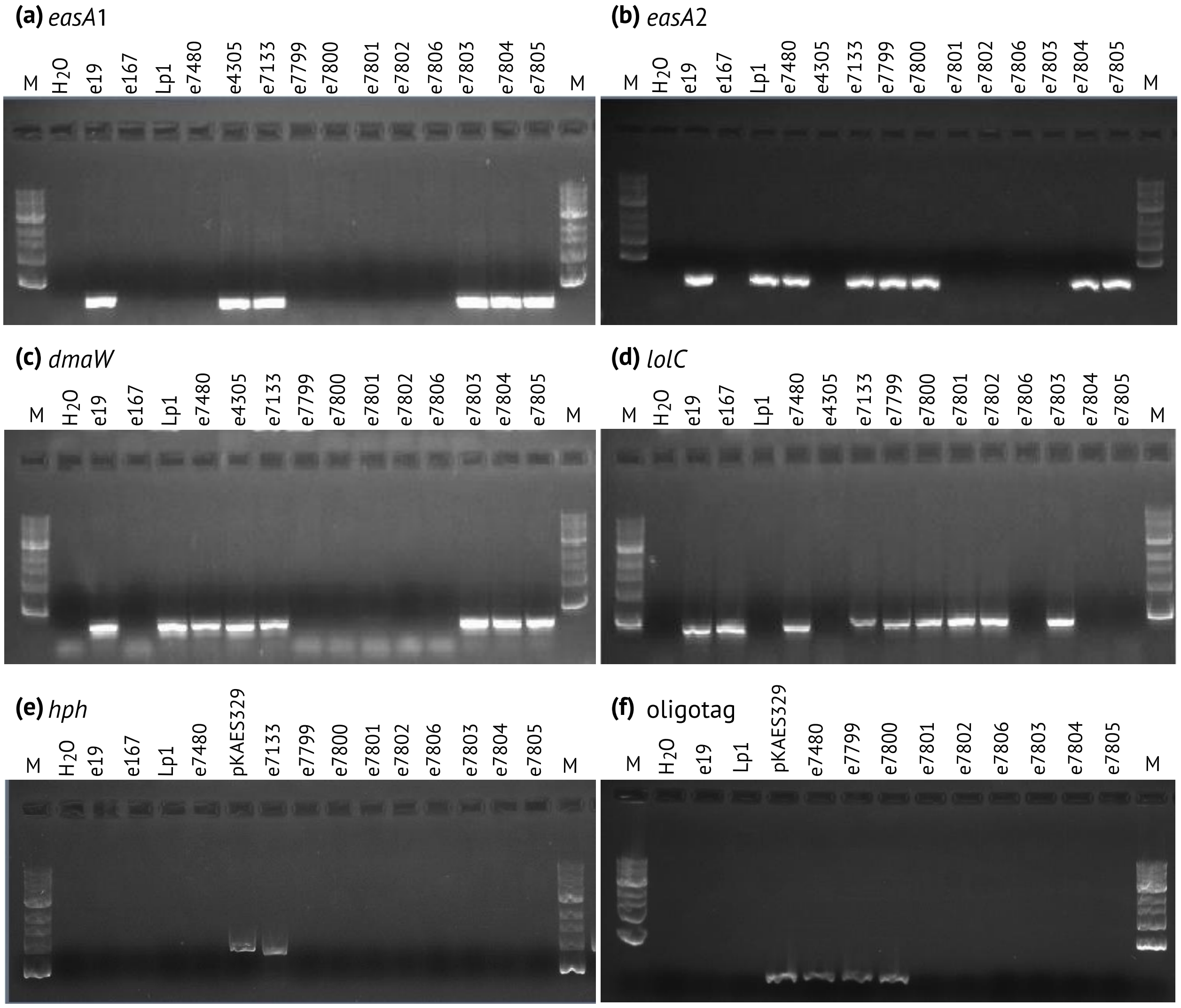

Figure S1. PCR tests of putative CRISPR/Cas9-mediated deletion mutants. (**a**) PCR tests with primers specific for *easA*1 from the ergot alkaloid gene cluster *EAS*1. (**b**) PCR tests with primers specific for *easA*2 from *EAS*2. (**c**) PCR tests for *dmaW* genes. (**d**) PCR tests for the *lolC* gene from the loline alkaloid (*LOL*) gene cluster. (**e**) PCR tests for the *hph* gene present on the selectable plasmids pKAES328 and pKAES329. (**f**) PCR tests for the oligotag present on the plasmids. Controls are: H_2O = no template PCR control; WT = *Epichloë coenophiala* e19, harboring *EAS1, EAS2* and *LOL* gene clusters; e167 = wild-type *Epichloë uncinata,* which lacks all *EAS* genes but harbors two *LOL* gene clusters; Lp1 = *Epichloë hybrida* Lp1, which has an *EAS*2 orthologue but no *EAS*1 or *LOL* cluster; e4305 = WT *Epichloë* sp. FaTG-4 which has an *EAS*1 orthologue but no *EAS*2 or *LOL* cluster; e7133 = a derivative of e19 that possesses *dmaW*1 but has an *hph* cassette in place of a partial deletion in *dmaW*2; e7480 = an *EAS*1-knockoff mutant of e19. Screened isolates (Table 3) are: putative *ΔdmaW*2 mutants of e7480 designated e7799 and e7800; putative *ΔEAS*1 *ΔEAS*2 mutants of e19 designated e7801 and e7802; a putative *ΔEAS*2 mutant

of *E. hybrida* Lp1 designated e7806; a putative *ΔEAS2* mutant of e19 designated e7803; and putative *ΔlolC* mutants of e19 designated e7804 and e7805.
